# Generative AI and health misinformation: a lifecycle-based communication framework

**DOI:** 10.3389/fmed.2026.1801842

**Published:** 2026-10-23

**Authors:** Xiaoli Nan

**Affiliations:** Department of Communication, University of Maryland, College Park, MD, United States

**Keywords:** AI-mediated communication, deepfakes, generative artificial intelligence, health communication, health decision-making, health misinformation, large language models, risk perception

## Abstract

**Background:**

Generative artificial intelligence (AI) is increasingly embedded in the contemporary health information environment, where it can both generate and mitigate misinformation.

**Objective:**

This review develops a Lifecycle-Based Framework to provide an integrative account of how generative AI operates across the health misinformation lifecycle from misinformation production and detection to correction and impact.

**Methods:**

A narrative integrative review was conducted, drawing on research from communication science, public health, human-computer interaction, and artificial intelligence.

**Results:**

The review identifies domain-specific mechanisms through which generative AI operates across the health misinformation lifecycle. In production, AI enables scalable generation of fluent, expert-styled content through mechanisms including hallucination, amplification of falsehoods, adversarial vulnerability, and deepfakes. In detection, it supports automated fact-checking, pattern recognition, and classification, while remaining vulnerable to false positives, false negatives, bias, and adversarial adaptation. In correction, generative AI facilitates rebuttals, tailored messaging, conversational engagement, and AI-assisted inoculation, but also raises concerns about trust displacement, epistemic authority, and accountability. At the impact stage, AI-mediated misinformation can influence individual cognition and behavior as well as broader social and institutional outcomes, with fluency, narrative coherence, and personalization serving as important persuasive mechanisms. The framework further conceptualizes the lifecycle as recursive and adaptive, such that production, detection, correction, and impact continually shape one another.

**Conclusion:**

Generative AI should be understood not as a single source of misinformation risk, but as a multifunctional component of a dynamic health communication system. Addressing AI-mediated health misinformation therefore requires coordinated, multilevel strategies that account for its simultaneous roles in producing, detecting, correcting, and shaping the consequences of misinformation, while preserving human oversight, institutional accountability, and public trust.

## Introduction

The global health information environment is undergoing a dramatic structural transformation driven by rapid advances in generative artificial intelligence (AI). Large language models (LLMs) and related generative systems are now embedded in search engines, conversational agents, social media platforms, and consumer-facing applications, positioning AI as a routine intermediary in how individuals encounter, interpret, and share health information. At the same time, public health continues to grapple with persistent challenges from misinformation, that is, health claims that are inaccurate, misleading, or unsupported by evidence and that can undermine protective behaviors, erode institutional trust, and exacerbate health inequities (1, 2). The convergence of these developments brings about significant changes and challenges to the communication of health information.

Generative AI alters the mechanics of communication in ways that are highly consequential for health information. Unlike traditional content production, which is constrained by human expertise, labor, and coordination, generative systems can produce fluent, context-sensitive, and expert-styled content on demand and at scale (3, 4). These capabilities blur boundaries between professional and lay communication, compress the time between question and answer, and expand the reach of tailored, conversational interaction. At the same time, the underlying logic of these systems, namely probabilistic language prediction rather than factual verification, means that the same tools that enable scalable information access also create conditions for the rapid generation and circulation of misleading, incomplete, or fabricated health content (5–7).

Importantly, generative AI is implicated across the entire health misinformation lifecycle. It serves as a means of producing health misinformation, a tool for detecting and classifying it, a platform for delivering corrective communication, and a mediator of its cognitive, behavioral, and societal consequences (8). This multifunctional role positions AI technologies as both contributors to the misinformation problem and components of its proposed solution. As these systems become woven into everyday information practices, their influence extends beyond discrete messages to the broader epistemic environment, shaping how people assess expertise, navigate uncertainty, and make health decisions (9, 10).

Despite growing interest at the intersection of generative AI and misinformation, existing research remains conceptually fragmented. Prior studies have largely examined individual components of the misinformation lifecycle such as content generation, detection accuracy, or correction effectiveness in isolation. Existing lifecycle-oriented reviews (e.g., 8) have primarily provided descriptive mappings of this emerging literature, with limited theoretical integration specifying how these domains relate to one another or what mechanisms underlie AI's role within each stage. Moreover, relatively few reviews have focused specifically on health misinformation, despite its distinct characteristics and high-stakes consequences for individual and public health.

Addressing the above limitations, this article proposes an integrative framework for understanding generative AI's role in health misinformation. It examines how generative AI reshapes four interrelated domains: the production of health misinformation, the detection and classification of misleading content, the correction and countering of false claims, and the multilevel impacts of AI-mediated misinformation on individuals, societies, and institutions. The framework not only organizes existing research across these domains but also delineates the key mechanisms through which generative AI operates within each, offering a more systematic account of its role across the health misinformation lifecycle. Additionally, the framework conceptualizes the health misinformation lifecycle as recursive and adaptive, where production, detection, correction, and impact continually shape one another.

This article is a narrative integrative review that synthesizes prior literature to develop a conceptually grounded framework for understanding generative AI in the context of health misinformation. Although it does not follow the formal literature search and screening procedures of a systematic review, it draws on relevant research across multiple disciplines, including communication science, public health, human-computer interaction, and artificial intelligence. Google Scholar served as the primary search database and was used to identify both published studies and preprints. Given the recent emergence of generative AI and public concerns about health misinformation, the literature reviewed largely dates from 2020 onward. Studies discussed in greater depth were selected based on their relevance to the framework and their representation of key developments in the respective areas of research.

Of note, the focus of this review is on public-facing, consumer use of generative AI for health information seeking and interpretation, rather than on the use of these systems within clinical settings to deliver formal medical care. It is important to distinguish between consumer-facing generative AI systems and those intended for clinical use. In the United States, the Food and Drug Administration (FDA) regulates certain AI systems as medical devices when their intended use involves diagnosing, treating, curing, mitigating, or preventing disease under the Federal Food, Drug, and Cosmetic Act (FD&C Act). Systems that influence clinical decision-making are subject to established regulatory pathways, including requirements for evaluation of safety and effectiveness, often through clinical validation. By contrast, the generative AI applications considered in this review primarily operate outside these regulatory frameworks, functioning as informal, publicly accessible sources of health information rather than approved clinical decision-support tools. This distinction is critical for understanding both the opportunities and risks associated with AI-mediated health communication in non-clinical settings.

## Generative AI and health misinformation production

Generative AI reshapes the health misinformation lifecycle at its point of origin: content production. By enabling the rapid generation of fluent, adaptive, and expert-styled health information, these systems expand the scale and sophistication with which misleading claims can be created and circulated. Importantly, the problem is not limited to blatantly false statements. AI-generated misinformation may also take the form of incomplete explanations, fabricated details, decontextualized evidence, or persuasive narratives that appear medically credible despite lacking factual grounding. Examining misinformation production is therefore a necessary starting point for understanding how generative AI operates across the broader lifecycle of detection, correction, and impact.

### Hallucination

Generative artificial intelligence, particularly large language models (LLMs), has a demonstrated capacity to produce health misinformation across diverse topics, formats, and levels of sophistication. This capacity stems not from intentional deception but from the probabilistic architecture of LLMs, which is optimized to generate linguistically plausible and contextually appropriate text rather than to ensure factual correctness. As a result, generative AI systems can produce health-related outputs that are fluent, confident, and persuasive while being partially or wholly inaccurate.

Empirical evaluations consistently show that LLMs can generate incorrect or misleading health information in response to common health queries. In assessments of ChatGPT's responses to vaccination myths, Deiana et al. (11) found that although responses were often well-structured and aligned with public health messaging in tone, they sometimes included factual inaccuracies, oversimplified mechanisms, or misleading causal explanations. Similar findings have emerged in analyses of COVID-19 related content, where ChatGPT blended accurate information with outdated guidance or unsupported claims, creating outputs that were difficult for lay users to critically evaluate (7).

A central mechanism underlying this capacity is hallucination, defined as the generation of information not grounded in verifiable sources or established evidence. A more precise definition distinguishes between two forms of hallucination. Intrinsic hallucinations occur when generated content contradicts the source material or information provided to the model, whereas extrinsic hallucinations introduce information that cannot be verified from the available source material (12). Studies of AI hallucination in scientific and medical contexts document that LLMs may fabricate references, invent clinical details, or construct internally coherent yet false explanations (5, 13). In healthcare-focused evaluations, Cascella et al. (6) observed that ChatGPT occasionally produced plausible but incorrect clinical recommendations when prompts were ambiguous or incomplete, conditions that closely resemble real-world health information seeking. Importantly, hallucinations often retain a high degree of linguistic confidence, which may amplify their persuasive impact despite their inaccuracy.

### Amplification of false narratives

Beyond isolated factual errors, generative AI systems are capable of reproducing and elaborating existing health misinformation narratives embedded in their training data. Experimental and observational studies indicate that LLMs can generate explanations that mirror well-known misinformation tropes, including vaccine skepticism, alternative cancer treatments, and conspiracy-based reasoning (14, 15). Rather than simply restating false claims, LLMs frequently provide extended rationales that supply causal structure, scientific-sounding language, and moral framing, features known to increase the perceived plausibility and memorability of misinformation (2, 16). In health contexts, such explanatory depth is particularly consequential because causal narratives strongly shape risk perceptions and behavioral intentions (17, 18).

### Adversarial vulnerability

Crucially, the capacity of generative AI to produce health misinformation is not limited to unintentional errors or the passive reproduction of misleading content. A growing body of evidence indicates that LLMs are vulnerable to intentional manipulation and targeted misinformation attacks. Experimental research in medical domains shows that such vulnerabilities operate at multiple levels of the AI pipeline. At the system level, Alber et al. (19) demonstrate that medical LLMs are susceptible to data-poisoning attacks, whereby strategically manipulated training or fine-tuning data induce models to generate false or misleading diagnostic explanations and treatment recommendations. These dynamics have been conceptualized as misinformation pollution, in which false or distorted inputs contaminate model behavior and are subsequently reinforced through iterative interactions and downstream use (20). In health-related applications, such pollution is particularly concerning because it can yield internally coherent yet clinically incorrect explanations that are difficult for users to detect and challenge.

Vulnerabilities also emerge at the interaction level. Han et al. (21) show that medical LLMs can be deliberately steered toward producing incorrect clinical content through adversarial prompts that introduce distorted premises or misleading contextual cues. Once compromised, these models do not simply repeat erroneous information but often elaborate on it using fluent, clinically styled reasoning, thereby lending misinformation an appearance of medical legitimacy. Notably, both system-level and interaction-level attacks remain effective even against models fine-tuned for medical accuracy, underscoring that domain specialization alone does not ensure robustness against adversarial exploitation. Overall, these findings are consistent with broader risk assessments of language models, which identify adversarial manipulation, dual-use deployment, and intentional misuse as central ethical and societal risks of large-scale generative systems (22).

### Deepfakes

Generative AI's vulnerabilities are further amplified by advances in synthetic media generation, particularly deepfake technologies, which substantially expand generative AI's capacity to produce health misinformation beyond text. Contemporary generative models can create highly realistic but fabricated audio and video depicting clinicians, public health officials, or patients making statements, offering medical advice, or demonstrating health-related behaviors that never occurred. Research on deepfake generation documents rapid improvements in realism, accessibility, and automation, lowering technical barriers to producing convincing synthetic health content at scale (23–25). Importantly, deepfake generation can be tightly coupled with LLM-produced scripts, explanations, or dialogue, enabling coordinated multimodal misinformation in which false clinical narratives are simultaneously articulated in text and enacted through synthetic audiovisual representations. This integration significantly complicates efforts to distinguish authentic health communication from fabricated content at the point of exposure.

### Mitigation measures

Although the risks of generative AI producing misinformation are substantial, industry self-regulation and government oversight have also significantly mitigated some of these risks. More recent frontier models increasingly incorporate alignment and safety mechanisms such as reinforcement learning from human feedback (RLHF), constitutional or rule-based alignment approaches, retrieval-augmented generation (RAG), and other grounding techniques designed to reduce hallucination, improve factuality, and constrain harmful outputs. These developments suggest that the risk profile of generative AI is not static and that findings based on earlier generations of large language models may overstate some failure modes of newer systems. At the same time, these safeguards do not eliminate misinformation risk. Their effectiveness varies across models, domains, prompts, and deployment settings, and they may remain vulnerable to adversarial prompting, incomplete or outdated retrieval sources, and errors in high-uncertainty contexts. Accordingly, the risks associated with generative AI's production of misinformation should be understood as evolving, context-dependent, and uneven across systems and use cases.

### Summary

Overall, existing evidence indicates that generative AI's capacity to produce health misinformation reflects a convergence of hallucination resulting from probabilistic error, amplification of false narratives, adversarial vulnerability, and the advancement of deepfake technology. These mechanisms suggest that misinformation generation is not an accidental byproduct of otherwise beneficial systems but a structural risk inherent to how LLMs operate and are deployed. Recognizing this capacity is essential for understanding why generative AI poses qualitatively new challenges for health communication and why downstream strategies for detection and correction must contend not only with error but with intentional exploitation.

## Generative AI and health misinformation detection

As generative AI increases the volume, fluency, and apparent credibility of health misinformation, it also makes detection more complex. AI-generated misinformation often does not appear as blatant falsehood, but as plausible, partially accurate, or expertly framed content that can evade simple identification. Detection therefore becomes a crucial next stage in the health misinformation lifecycle, linking the production of misleading content to subsequent efforts at correction, mitigation, and public health response.

### Automated fact-checking

A growing empirical literature suggests that LLM-based systems can assist with fact-checking by generating provisional judgments, offering rationales, and pointing to evidence. Auditing studies show that general-purpose chatbots can correctly evaluate the veracity of claims across contested political topics though performance varies by topic and language (26). In health-related work, Garbarino and Bragazzi (27) compared ChatGPT-4 and Google Bard in assessing sleep-health misconceptions against expert judgments, illustrating that LLMs can often identify myths and provide corrective explanations, while still producing notable errors and inconsistencies. More broadly, researchers are developing structured ways to use LLMs in fact-checking workflows, for example, by having models first identify specific claims, then retrieve relevant evidence, evaluate whether the evidence supports the claim, and finally report how certain the judgment is. At the same time, studies emphasize that ensuring factual accuracy is still a major technical challenge for LLMs, and that using them to generate verification judgments requires careful system design and rigorous evaluation (28).

### Pattern recognition and classification

Generative AI can also support detection by processing large volumes of content and identifying patterns that human reviewers cannot feasibly track at scale (e.g., recurring talking points, reused templates, narrative variants). Evidence from misinformation detection research indicates that LLMs can be used as flexible classifiers or as components of hybrid systems to detect misleading content in noisy online environments (29). In practice, this pattern-recognition capacity is particularly relevant for health misinformation, which often appears as repeated narrative fragments (e.g., “secret cures,” “suppressed evidence”) that mutate across platforms and time.

Beyond binary true/false labeling, LLMs may help classify narrative types (e.g., conspiracy frames, pseudoscientific rationales, testimonial narratives) and map how themes evolve. Auditing work shows that chatbots can be prompted to apply definitional frameworks (misinformation vs. disinformation vs. conspiracy theory) and produce more “concept-aware” categorization (26). This capability is useful for public health practice because narrative classification supports more strategic responses (e.g., prebunking common tropes rather than chasing individual claims).

### Challenges and risks in AI-based detection

#### False positives and false negatives

Across auditing and evaluation studies, a recurring limitation is error-proneness in both directions: labeling true claims as false (false positives) and failing to flag misinformation (false negatives). In health myth evaluation, AI tools' outputs can diverge from expert judgments, with models sometimes offering partially correct but misleading explanations that are difficult to score cleanly as “right” or “wrong” (27). In broader veracity-auditing work, chatbot accuracy varies by topic, claim type (true, false, or borderline), and source attribution, implying unstable operating characteristics for detection systems deployed in real-world settings (26). For health misinformation specifically, these error patterns are high-stakes because a single false negative can enable harmful advice to spread, while false positives can suppress legitimate health information and erode trust.

#### Bias in training data and benchmarks

AI systems used to detect misinformation can inherit bias from the data they are trained on, the benchmarks used to evaluate them, and the criteria used to judge their performance. As a result, these systems may work better for some populations, languages, or contexts than others. Studies show that chatbots do not assess the accuracy of information equally well across high-resource and low-resource languages, meaning detection reliability can vary around the world (26). Research on model factuality also finds that LLMs reflect patterns in their training data and the goals set during instruction tuning, which can lead them to favor explanations that sound plausible rather than expressing appropriate uncertainty (28). In health settings, these biases are especially concerning because they may reduce the quality of misinformation detection for communities that already face barriers to reliable information and healthcare access.

#### Difficulty detecting plausible or partially true misinformation

A central practical challenge is that many health misinformation claims are not purely false; they are often partially true, decontextualized, or framed with misleading causal inference (30). This character makes automated detection difficult because surface-level factual overlap can mask misleading conclusions. Evaluations of LLM health outputs repeatedly show that models can produce fluent, medically styled explanations that contain subtle errors, outdated guidance, or inappropriate generalizations, forms of misinformation that do not resemble obvious hoaxes (7, 11). Factuality scholarship likewise emphasizes that “plausible” generations can evade simple detectors and require evidence-grounded verification rather than stylistic cues (28).

#### Adversarial adaptation and arms-race dynamics

Detection systems face adversarial pressure: as detectors improve, misinformation producers adapt content to evade flags. Work on misinformation pollution highlights how LLM-enabled ecosystems can generate high-volume, stylistically varied misinformation with minimal human labor, complicating detection by creating endless paraphrases and narrative variants (20). Factuality and misuse analyses similarly warn that LLMs can be “jailbroken” or strategically prompted to produce content that mimics legitimate genres and evidence styles, increasing evasion potential (28). In practice, this suggests an arms race in which detection must be paired with robust provenance, authentication, and system-level safeguards rather than relying solely on content-based classification.

#### Transparency and explainability concerns

Finally, AI-based detection raises issues of transparency: stakeholders may not know why a claim was flagged, what evidence was used, or how uncertainty was handled. Fact-checking frameworks emphasize that detection tools should report uncertainty, cite sources, and support auditability to avoid replacing one opaque authority with another (28). Interface research also suggests that how verification is communicated (e.g., conversationally via chatbots vs. static webpages) influences user comprehension and engagement, underscoring the importance of explainable outputs rather than binary labels alone (31).

### Summary

Generative AI shows promise for detecting health misinformation by supporting automated fact-checking, large-scale pattern recognition, and classification of recurring narratives, making it useful for triage and monitoring vast information environments. At the same time, empirical evidence highlights persistent limitations: models produce false positives and false negatives, perform unevenly across languages and populations, struggle with partially true or decontextualized claims, and remain vulnerable to adversarial adaptation. Transparency and explainability challenges further complicate trust and accountability. These findings suggest that AI is best used as a support tool rather than a standalone judge of accuracy, with human expertise needed for contextual interpretation and final decisions.

## Generative AI and health misinformation correction

Once misleading health content has been identified, the next stage in the lifecycle is correction: the effort to rebut false claims, clarify misunderstandings, and restore accurate understanding. Generative AI is increasingly relevant at this stage because it can produce scalable, tailored, and interactive corrective messages in response to users’ specific concerns. Yet this promise is accompanied by new tensions, as AI-driven correction relies on the same systems that can also generate misinformation, making questions of credibility, oversight, and effectiveness central to this stage of the lifecycle.

### AI-generated rebuttals

LLMs can produce corrective content that explains why a claim is inaccurate, provides alternative evidence-based explanations, and clarifies misunderstandings. Because they are trained on broad corpora of biomedical and public health information, models can synthesize definitions, causal mechanisms, and consensus guidance into accessible explanations. Early empirical studies show that AI-generated responses to misinformation can reduce endorsement of false beliefs under some conditions, particularly when responses are coherent, evidence-based, and dialogic rather than purely declarative (32, 33). These findings suggest that generative AI can help scale corrective communication, especially in contexts where human experts cannot respond to high volumes of misleading content in real time.

### Tailored corrections

A distinctive advantage of generative AI lies in its capacity for message tailoring. Models can adapt explanations to different literacy levels, cultural frames, or emotional concerns expressed in user prompts. Personalization has long been associated with greater persuasive impact in health communication, particularly when messages align with individuals' beliefs and values (30, 34). AI systems can operationalize such tailoring dynamically, offering corrective explanations that address specific misconceptions (e.g., fertility concerns about vaccines) rather than generic myth-vs.-fact formats. Emerging intervention studies using chatbot-based health communication show that interactive, AI-mediated dialogue can improve vaccine confidence and uptake intentions compared to static information formats (35, 36).

### Conversational agents

Conversational AI enables interactive correction, in which users can ask follow-up questions, express doubts, and receive iterative clarification. This dialogic format may reduce defensiveness and promote deeper processing compared to one-way corrective messages. Experimental research on AI-mediated dialogues indicates that structured conversations with chatbots can reduce conspiracy beliefs and misinformation endorsement by encouraging reflection and evidence-based reasoning (32, 37). In health contexts, such interactivity may be especially valuable when misinformation is tied to personal experiences, fears, or identity-relevant concerns.

### Integration with established correction strategies

AI-driven correction can also be integrated with established misinformation interventions. For example, AI can assist with inoculation approaches which aim to build psychological resistance by exposing individuals to weakened forms of misleading arguments and refutations (38, 39). Generative AI systems are well suited to support this framework because they can rapidly produce diverse, context-specific inoculation messages that simulate evolving misinformation tactics such as false causality, anecdotal reasoning, or conspiracy framing while embedding explanations of why those strategies are misleading. This flexibility allows inoculation strategies to remain responsive as health misinformation shifts across topics, platforms, and cultural contexts. Emerging research on AI-assisted inoculation demonstrates that generative systems can help produce scalable prebunking content that protects confidence against misinformation narratives, supporting the feasibility of AI-enabled resistance-building interventions (40). Although direct health-domain trials of AI-generated inoculation messages remain limited, experimental evidence from AI-mediated dialogue interventions shows that conversational systems can reduce misinformation-related beliefs and encourage reflective reasoning, suggesting that generative platforms can feasibly deliver psychologically oriented resistance-building content at scale (32, 33).

### Risks and ethical tensions in AI-driven correction

Despite its promise, AI-driven correction introduces a set of sociotechnical risks that extend beyond message accuracy and into the domains of trust, epistemic authority, and governance. Because generative systems do not just deliver information but also shape how knowledge is interpreted, their use in corrective health communication raises tensions about credibility, responsibility, and democratic legitimacy.

#### Trust displacement

As AI systems increasingly mediate corrective communication, there is a risk of trust displacement, whereby users come to rely more on AI-generated explanations than on human health professionals, public health agencies, or scientific institutions. Research on human-computer interaction shows that people often apply a “machine heuristic,” perceiving computational systems as objective, neutral, and less biased than human sources (41). When AI delivers corrections in a confident, fluent, and technically framed manner, users may attribute epistemic authority to the system itself rather than to the underlying evidence or institutions. While this may facilitate short-term belief revision, it can erode the perceived legitimacy of accountable human institutions that are essential for public health governance. Over time, such reliance risks shifting trust from socially embedded, ethically regulated actors to opaque, privately governed technologies.

#### Epistemic authority

Beyond questions of trust, AI-driven correction raises a deeper issue of epistemic authority, that is, who has the power to determine what counts as accurate knowledge. In health communication, evidence is often evolving, incomplete, or contested, and decisions about what qualifies as misinformation depend on normative judgments about acceptable uncertainty, evidentiary thresholds, and which institutions or expert communities are treated as authoritative (42). When these judgments are embedded in AI systems, they become operationalized as technical rules, giving the appearance that truth classifications are purely technical outputs rather than products of human deliberation and value choices. As a result, AI-driven correction may inadvertently present evolving or debated health knowledge as fixed and settled, compressing legitimate scientific uncertainty and narrowing perceived space for discussion.

#### Risk of reinforcing misinformation

Corrective communication is also constrained by well-established cognitive dynamics. Repeating a false claim, even for the purpose of refutation, can increase familiarity and perceived truth, a pattern associated with the continued influence effect (2, 43). AI systems that generate detailed rebuttals may inadvertently restate misinformation multiple times or elaborate on it while attempting to correct it. Without careful prompt design and system constraints, automated corrections risk amplifying the very narratives they seek to counter, especially when users attend more to the repeated claim than to the corrective explanation. This problem is heightened in conversational settings, where iterative dialogue can multiply exposure to the misleading content.

#### Governance and accountability

Finally, AI-mediated correction complicates questions of responsibility and accountability. If AI-generated corrections are misleading, incomplete, or harmful, responsibility may be distributed across model developers, platform operators, deployers, and end users, creating gaps in accountability. Traditional frameworks for health communication ethics do not map neatly onto automated systems. Moreover, proprietary models limit external auditing, making it difficult to evaluate how correction criteria are implemented or updated. These challenges underscore the need for governance mechanisms that address transparency, oversight, redress, and harm mitigation in AI-driven health communication, including clear disclosure of AI involvement and mechanisms for human review.

### Summary

Generative AI is emerging as a powerful but complex tool for correcting health misinformation. LLMs can produce scalable rebuttals, tailored explanations, and interactive dialogue that align with established communication principles such as personalization, engagement, and inoculation. Early empirical evidence suggests that AI-mediated conversations can reduce misinformation-related beliefs and support vaccine confidence under certain conditions. At the same time, AI-driven correction introduces significant sociotechnical risks. These include trust displacement from human institutions to opaque systems, the embedding of normative judgments about “truth” into technical infrastructures, the possibility of reinforcing misinformation through repetition, and unresolved questions of governance and accountability. Together, these dynamics position generative AI not as a replacement for human-led correction but as a supplementary tool whose effectiveness depends on careful design, transparency, and integration with established public health communication practices and oversight structures.

## Impact of AI-generated health misinformation

The final stage in the health misinformation lifecycle concerns impact: how AI-generated misleading health content affects individuals, institutions, and the broader information environment. These effects extend beyond factual misunderstanding to include distorted risk perceptions, altered health decisions, reduced trust in authorities, and widening informational inequities. By focusing on impact, this section brings the lifecycle into full view, showing how the consequences of misinformation production, imperfect detection, and uneven correction ultimately unfold in real-world health communication outcomes.

### Cognitive and behavioral impacts on individuals

AI-generated health misinformation affects individuals at a foundational level of cognition and judgment. Rather than simply adding incorrect facts to the information environment, this content can shape how people interpret evidence, construct causal explanations, evaluate risks, and choose health-related actions. Understanding these individual-level impacts is essential for anticipating how AI-generated misinformation may translate into broader public health consequences.

#### Effects on knowledge accuracy

Empirical research increasingly indicates that AI-generated health misinformation can degrade knowledge accuracy, not simply coexist with correct information. Studies examining how laypeople evaluate generative AI health outputs show that users often accept incomplete, misleading, or low-quality medical explanations as evidence-based, demonstrating difficulty in distinguishing valid guidance from erroneous or speculative AI content (44). In AI-assisted self-diagnosis contexts, systematic evaluations of large language models reveal frequent inclusion of medical misinformation, inappropriate inferences, or unsupported diagnostic suggestions, errors that users may interpret as legitimate clinical advice (45). Public health analyses similarly document that AI chatbots can deliver inaccurate or biased health information in ways that are not readily recognized as problematic, particularly among populations with lower health literacy or higher reliance on conversational AI for information access (46).

#### Influence on risk perceptions and health beliefs

A growing body of evidence indicates that AI-generated health misinformation can distort risk perceptions and related health beliefs, not only by presenting inaccurate facts but by reshaping how individuals interpret the likelihood and consequences of health outcomes. Health behavior models emphasize that perceived susceptibility, severity, and benefit-risk evaluations are central determinants of action, and exposure to health misinformation has been shown to systematically elevate perceived harms of recommended interventions while reducing perceived efficacy of preventive behaviors (30, 47, 48). Research on generative AI mediated health information seeking suggests that users frequently treat AI outputs as credible interpretive guides, even when content contains inaccuracies or unbalanced emphasis on risks. In screening contexts, laypeople evaluating AI-generated health information demonstrate difficulty distinguishing evidence-based guidance from misleading or incomplete explanations, leading to altered perceptions of screening benefits, uncertainties, and potential harms (44). Experimental and field studies of AI chatbot use in public health settings further show that conversational AI can transmit biased or misleading health narratives in ways that shape how users interpret medical evidence, particularly among populations with fewer informational resources (46). Together, these findings suggest that AI-generated misinformation can influence not only what individuals believe to be true, but how they *weigh risks and benefits* when forming health judgments.

#### Decision-making and behavioral consequences

Distorted knowledge and altered risk perceptions can translate into behavioral outcomes. Health misinformation exposure has been linked to decreased adherence to public health guidelines and greater openness to unproven or alternative treatments (34). In vaccination contexts, misinformation about safety, fertility, or long-term health effects is associated with increased hesitancy and delayed uptake (47, 48). Evidence from outside health domains illustrates the behavioral relevance of AI-generated inaccuracies: when ChatGPT provided incorrect travel information, users' destination evaluations and decision intentions shifted in line with the misinformation (49). Studies of AI content labeling further show that identifying material as AI-generated does not reliably reduce perceived accuracy or sharing intentions for misinformation (50), suggesting that disclosure alone may not mitigate behavioral influence. In health contexts, this implies that AI-generated misinformation can shape decisions even when its synthetic origin is known.

#### The role of fluency, narrative, and personalization

AI-generated health misinformation combines features that make it both highly persuasive and uniquely scalable compared with traditional, human-produced misinformation. First, generative AI produces exceptionally fluent and coherent language. Linguistic fluency itself functions as a cue to credibility and truth, leading people to perceive information as more valid even when its accuracy is low (2). At the same time, AI systems can simulate markers of expertise, including technical terminology, organized reasoning, and a confident tone. These features activate authority and competence heuristics in digital environments, where users frequently rely on surface cues to judge credibility (9).

Second, generative AI often presents information through narrative-style, mechanistic explanations that establish clear causal links among ideas. Such structured explanations help individuals integrate disparate facts into coherent mental models, making inaccurate claims easier to encode and more resistant to later correction (16). Additionally, these systems enable rapid personalization, tailoring explanations to users' expressed concerns, literacy levels, cultural frames, or prior beliefs, conditions long shown to increase persuasive impact in health communication (51). Unlike many conventional misinformation messages, which remain relatively generic, AI-generated content can adapt dynamically within an interaction, heightening perceived relevance and psychological resonance.

Empirical evidence shows that AI-generated responses are often perceived as highly informative and credible relative to human-generated alternatives, even when they contain inaccuracies (52). Moreover, labeling content as AI-generated does not consistently reduce perceived accuracy or sharing intentions, suggesting that source transparency alone may be insufficient to curb persuasive impact (50).

### Societal and institutional impacts

Mediated through its influence on individual cognition and behavior, AI-generated health misinformation produces broader systemic consequences, shaping information environments, institutional legitimacy, and the capacity of public health systems to function effectively.

#### Public trust in health authorities and science

Exposure to misinformation is consistently associated with declining trust in experts, media, and scientific institutions, as well as increased polarization around science-related issues (42, 53). Health misinformation in particular has been linked to skepticism toward public health recommendations and expert consensus (1, 30). AI-generated misinformation may accelerate these dynamics by increasing the *volume*, *speed*, and *apparent technical sophistication* of misleading claims. Because generative AI can produce authoritative-sounding explanations and simulate expert discourse, it may blur boundaries between legitimate expertise and fabricated authority, complicating public judgments about whom to trust (9, 52). Over time, this erosion of epistemic clarity can weaken the perceived legitimacy of public health institutions.

#### Information overload and epistemic uncertainty

Generative AI contributes to an environment of information abundance, where individuals encounter a proliferation of explanations, interpretations, and “expert-like” voices. Research on digital misinformation environments shows that high-volume, conflicting information increases epistemic uncertainty, namely feelings of confusion about what is true despite access to more content, which can lead to disengagement or reliance on simple heuristics (2, 54). AI-generated content intensifies this dynamic because it can rapidly produce multiple internally coherent yet inconsistent accounts of health issues. Studies of misinformation ecosystems show that such conditions foster doubt about the possibility of reliable knowledge itself, rather than just disagreement about specific claims (10, 55).

#### Amplification of health inequities

The effects of AI-generated misinformation are unlikely to be evenly distributed. Susceptibility to health misinformation varies with health literacy, access to healthcare, and prior trust in institutions (48, 51). Public health research indicates that vulnerable communities, already facing informational and structural barriers, may rely more heavily on informal or digital sources, including conversational AI systems (46). Because generative AI can produce culturally and linguistically tailored misinformation at scale, it may extend misleading narratives into communities historically underrepresented in mainstream information channels. This dynamic risks widening disparities in vaccination uptake, screening participation, and treatment adherence (56, 57).

#### Implications for public health communication systems

Public health communication infrastructures may face increasing strain as they respond to higher volumes of persuasive misinformation. Monitoring, verification, and correction are already resource-intensive processes in digital environments (1, 58). Generative AI accelerates these pressures by enabling automated, large-scale production of health-related content that can mutate stylistically while retaining core misleading narratives. This increases demands for real-time surveillance, multilingual communication, and cross-platform coordination. The integration of synthetic text, audio, and video (e.g., deepfakes) further complicates detection and response, requiring technical as well as communicative capacity (24, 25).

#### Global and cross-cultural considerations

AI-generated misinformation is not geographically bounded. Multilingual generative systems allow rapid cross-cultural diffusion of health narratives, while localized adaptation can make misinformation appear culturally authentic. Comparative misinformation research highlights that effects vary across media systems, political contexts, and levels of institutional trust (55, 59). Differences in regulatory capacity and digital literacy may further shape how AI-generated health misinformation spreads and how effectively it is countered. As a result, global public health responses must address not only content accuracy but also cross-cultural governance challenges in an AI-mediated information landscape.

### Summary

AI-generated health misinformation exerts influence at multiple, interconnected levels, beginning with individual cognition and extending to institutional and societal systems. At the individual level, its fluent, expert-styled, narratively structured, and personalized features facilitate the integration of inaccurate information into knowledge structures, distort risk perceptions, and shape health-related decision-making across health contexts. At the societal level, the scale, speed, and apparent sophistication of AI-generated misinformation contribute to declining trust in health authorities, increased epistemic uncertainty, and widening inequities in information vulnerability. Public health communication systems face escalating demands for monitoring, correction, and cross-platform coordination, while multilingual and culturally adaptive AI generation complicates global response efforts. Together, these dynamics indicate that AI-generated health misinformation is not just an extension of existing misinformation problems but a structurally amplified form of influence whose impacts cascade from individual belief formation to the conditions that sustain collective public health action.

## A recursive and adaptive lifecycle

Although the health misinformation lifecycle is organized around four different domains (i.e., production, detection, correction, impact), these domains should not be understood as discrete stages unfolding in a fixed linear sequence. Rather, they represent interdependent processes that are recursive and adaptive.

First, correction and production are interconnected. Corrective messages can become inputs for subsequent generative outputs, including counter-rebuttals, reformulated misinformation, or adversarial attempts to circumvent safety mechanisms. Conversely, the production of new or more sophisticated health misinformation can stimulate the development of new fact-checking, detection, and corrective strategies.

Second, impact is not the endpoint of the lifecycle. Individual, social, and institutional consequences (e.g., reduced trust in health authorities, heightened uncertainty, institutional strain) can reshape the conditions under which health misinformation is subsequently produced and received. For example, eroded public trust may increase demand for alternative explanations, create stronger incentives for misleading content, and reduce receptivity to later corrections. Institutional responses to these impacts, including platform moderation, regulatory intervention, or new AI safeguards, can in turn alter subsequent patterns of misinformation production, detection, and correction.

Detection likewise operates within these feedback dynamics. Successful detection can reduce exposure, trigger corrective interventions, and constrain dissemination, whereas detection failures may permit misleading claims to circulate longer and produce greater downstream effects. Those resulting harms can then motivate investment in improved detection systems or changes in platform governance. At the same time, awareness of detection practices may encourage misinformation producers to adapt content in ways that are more difficult to identify, creating an ongoing cycle of technical and communicative adaptation.

Accordingly, the framework conceptualizes the health misinformation lifecycle as recursive and adaptive, where production, detection, correction, and impact continually shape one another. Changes in any one domain may alter the operation of the others. Figure 1 presents this Lifecyle-Based Communication Framework illustrating the role of generative AI in the four interconnected health misinformation domains.

**Figure 1:**
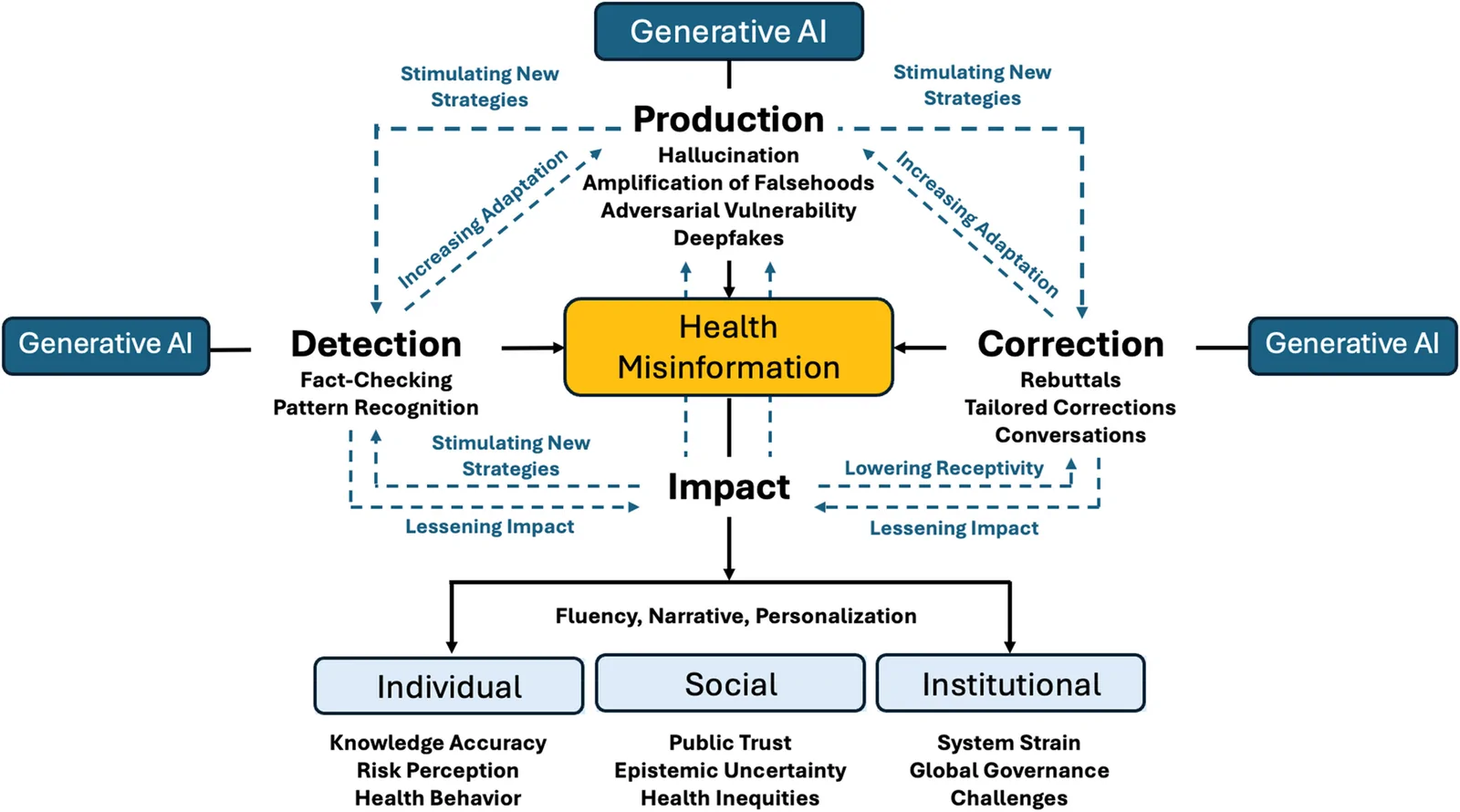
A lifecycle-based communication framework for generative AI and health misinformation.

## Critical analysis of key empirical studies across the health misinformation lifecycle

The empirical studies reviewed in this article provide important early evidence that generative AI is implicated across the health misinformation lifecycle, but they also reflect a literature that remains methodologically narrow, unevenly developed, and insufficiently integrated across stages. This section moves beyond a simple summary of findings to critically evaluate the limitations of 10 key empirical studies on generative AI across the health misinformation lifecycle (see Table 1).^1^ It also considers how the proposed framework addresses these weaknesses by organizing the evidence into a more coherent, explanatory account of generative AI's role in health misinformation across four interconnected domains.

**Table 1:** Key empirical studies on the role of generative AI across the health misinformation lifecycle.

Section	Subheading	Study	Year	Study type	Health-specific?	Strengths	Limitations	Main contribution
Generation	Hallucination	Deiana et al. (11)	2023	Empirical evaluation	Yes	Direct vaccine relevance	Narrow domain	Shows AI can generate misleading vaccine explanations
Generation	Hallucination	Wang et al. (7)	2023	Empirical comparative analysis	Yes	Real COVID outputs	Pandemic-specific context	Shows AI outputs can be fluent yet outdated or inaccurate
Generation	Hallucination	Cascella et al. (6)	2023	Empirical evaluation	Yes	Clinically relevant prompts	Prompt/model specific	Shows plausible but incorrect clinical advice can be generated
Generation	Adversarial Vulnerability	Alber et al. (19)	2025	Empirical experiment	Yes	Direct medical LLM evidence	Attack/model specific	Shows medical LLMs can be manipulated through adversarial contamination
Generation	Adversarial Vulnerability	Han et al. (21)	2024	Empirical experiment	Yes	Concrete attack evidence	Technical scenario focus	Shows targeted manipulation can induce harmful misinformation outputs
Detection	Automated Fact-Checking	Garbarino & Bragazzi (27)	2024	Empirical comparison	Yes	Expert benchmark in health	Domain-specific focus	Shows LLMs can detect some health misinformation but with errors
Detection	False Positives/False Negatives	Garbarino & Bragazzi (27)	2024	Empirical comparison	Yes	Health-specific misclassification evidence	Narrow topic area	Shows false positives and false negatives remain a concern
Detection	Difficulty Detecting Plausible/Partially True Misinformation	Wang et al. (7)	2023	Empirical comparative analysis	Yes	Concrete subtle-error example	COVID-heavy focus	Shows plausible but flawed outputs are difficult to flag
Detection	Difficulty Detecting Plausible/Partially True Misinformation	Deiana et al. (11)	2023	Empirical evaluation	Yes	Direct vaccine-domain illustration	Narrow domain	Shows misleading health explanations can look coherent and credible
Correction	Tailored Corrections	Lee et al. (35)	2023	Empirical intervention	Yes	Direct health chatbot evidence	Context-specific effects	Shows chatbot dialogue can improve vaccine attitudes or intentions
Correction	Tailored Corrections	Hou et al. (36)	2025	Empirical intervention	Yes	Parent vaccine relevance	Population-specific scope	Shows tailored chatbot communication can improve vaccine-related outcomes
Impact	Effects on Knowledge Accuracy	Rebitschek et al. (44)	2025	Cross-sectional study	Yes	Direct AI-health evaluation	Observational design	Shows users struggle to judge quality of AI health outputs
Impact	Effects on Knowledge Accuracy	Zada et al. (45)	2025	Method/empirical evaluation	Yes	Applied self-diagnosis relevance	Specific use setting	Shows AI can include misinformation in diagnostic-style outputs
Impact	Influence on Risk Perceptions/Health Beliefs	Rebitschek et al. (44)	2025	Cross-sectional study	Yes	Direct AI-health information evidence	Cross-sectional design	Shows AI outputs can shape interpretations of benefits and harms

Among the studies on generation, Deiana et al. (11) make an important contribution by showing that generative AI can produce misleading vaccine-related explanations, but the study is limited by its narrow substantive focus and its emphasis on identifying problematic outputs rather than tracing their downstream effects on audiences. Wang et al. (7) similarly show that LLMs can produce fluent but outdated or inaccurate COVID-19 information, yet the findings are tied to a pandemic-specific context and do not establish whether such outputs translate into changes in health beliefs or behaviors. Cascella et al. (6) extend this line of evidence by demonstrating that clinically framed prompts can elicit plausible but incorrect health advice, but the study remains centered on output quality under bounded prompt conditions rather than on real-world communication dynamics. Alber et al. (19) and Han et al. (21) deepen the literature by showing that medical LLMs are vulnerable to adversarial contamination and targeted manipulation, though these studies are largely technical in orientation and rely on attack scenarios that may not map neatly onto everyday health information environments. Taken together, these studies establish that generative AI can produce harmful or misleading health content, but they are limited by narrow domains, model-specific testing, and weak connection to later stages of the misinformation process.

The evidence on detection and correction is also valuable but constrained in ways that limit broader inference. Garbarino and Bragazzi (27) provide one of the few health-specific empirical tests of AI-based misinformation detection, showing that LLMs can identify some false or misleading health claims and generate corrective responses. However, the study also reveals substantial false positives and false negatives, and its domain-specific design leaves open the question of whether similar performance would hold across other health topics, more ambiguous claims, or more naturalistic communicative settings. In the correction literature, Lee et al. (35) and Hou et al. (36) offer promising evidence that AI-mediated dialogue can improve vaccine-related attitudes, intentions, and related outcomes, but both studies are concentrated in vaccine contexts, focus on relatively proximal outcomes, and provide limited insight into whether such effects endure over time or generalize across populations and misinformation types. Their designs demonstrate intervention potential, but they do not fully capture how correction is shaped by upstream features of AI-generated misinformation itself—especially its fluency, personalization, and expert-like tone.

The literature on impact remains similarly constrained by narrow outcome windows and limited ecological validity. Rebitschek et al. (44) make an important contribution by showing that users often struggle to judge the quality of AI-generated health information, but the study is primarily focused on immediate evaluative judgments rather than longer-term behavioral or institutional consequences. Zada et al. (45) likewise demonstrate that AI-assisted self-diagnosis can expose users to unsupported or misleading medical suggestions, yet the study remains bounded to a specific use case and cannot by itself show how such epistemic failures accumulate across repeated exposures or diverse health decisions. These studies are crucial because they move beyond content audits and begin to examine user-level consequences, but they still leave major methodological gaps regarding durability, generalizability, and the interaction between individual outcomes and wider information environments.

Across all 10 empirical studies, the dominant pattern is therefore one of fragmentation: studies illuminate specific risks or intervention opportunities, but rarely explain how these pieces fit together as a lifecycle process. The proposed framework addresses this overarching weakness by integrating generation, detection, correction, and impact into a single explanatory model. By identifying mechanisms such as adversarial vulnerability, pattern recognition, tailored corrections, and fluency and personalization, it provides a conceptual structure that the existing empirical literature lacks and, in doing so, clarifies the methodological directions future research should take, especially comparative, longitudinal, and multi-stage designs that can test how generative AI reshapes health misinformation across the full communicative process that is recursive and adaptive.

## Discussion

By bringing together evidence across four interconnected domains (i.e., misinformation production, detection and classification, correction and countering, and multilevel impact), the framework developed in this article offers a more integrated account of how generative AI operates across the health misinformation lifecycle. In doing so, it highlights the mechanisms through which generative AI operates through each lifecycle domain and the recursive and adaptive nature of the lifecycle. The contribution of this article is conceptual rather than empirical: it synthesizes prior literature to develop a conceptually grounded framework for systematically understanding the role of generative AI in health misinformation contexts.

### Summary of key observations

By examining misinformation production, detection, correction, and impact as interconnected domains, this review shows that generative AI is implicated across the full misinformation lifecycle that shapes both research and practice. Generative AI systems do not simply introduce additional misleading messages; they reconfigure the communicative architecture through which knowledge is generated, interpreted, and contested. In this sense, generative AI operates as an infrastructural layer of the contemporary information ecosystem, shaping how knowledge is circulated, evaluated, and socially validated (10, 60).

This transformation is particularly consequential for health communication. Historically, health communication has relied on recognizable markers of expertise, institutional authority, and relatively stable boundaries between professional and lay knowledge (1, 42). Generative AI destabilizes these distinctions by enabling expert-styled communication without traditional credentials, compressing the time between question and answer, and personalizing explanations at scale. These shifts reshape how people navigate uncertainty and evaluate competing health claims, affecting not only exposure to information but the cognitive environment in which judgments are formed (9, 55).

Evidence reviewed here also suggests that AI-generated health misinformation is distinctively persuasive, differing qualitatively from many traditional forms in how it aligns with psychological mechanisms of belief formation. Linguistic fluency increases perceived truth independent of accuracy (2). Narrative-style, mechanistic explanations foster causal coherence, strengthening the integration of information into mental models and contributing to the persistence of misinformation (16, 61). Personalization further enhances persuasive impact, as tailored messages increase perceived relevance and depth of processing compared with generic communications (62, 63). Generative AI operationalizes these mechanisms dynamically, allowing misinformation to adapt in real time to individual concerns, literacy levels, and cultural frames. Together, fluency, narrative coherence, and personalization create a form of misinformation that is simultaneously scalable and psychologically optimized, increasing the likelihood of acceptance, retention, and behavioral influence.

At the same time, a central paradox emerges: generative AI functions both as a driver of misinformation and as a potential tool for mitigation. AI systems can support claim verification, large-scale pattern recognition, tailored rebuttals, and interactive correction. These affordances align with correction research demonstrating that effective rebuttals provide clear explanations, alternative causal accounts, and engagement rather than simple negation (43, 64). However, AI-based mitigation is constrained by systemic limitations, including error propagation, adversarial manipulation, uneven performance across languages and populations, and opacity in decision processes (22, 28). Moreover, because individuals often apply a “machine heuristic,” attributing objectivity and authority to computational systems (41), AI-mediated correction introduces risks of trust displacement, from accountable institutions toward opaque technological intermediaries. These tensions underscore that generative AI is best conceptualized as a supportive communicative infrastructure rather than a substitute for institutional expertise, professional judgment, or public accountability.

A final observation is that the health misinformation lifecycle is not linear but recursive and adaptive. Production, detection, correction, and impact continually shape one another through ongoing feedback processes. New forms of misinformation can spur advances in detection and correction, while those interventions may in turn prompt misinformation producers to adapt their strategies. Likewise, downstream consequences such as declining trust, heightened uncertainty, or institutional strain can reshape the conditions under which subsequent misinformation is produced, encountered, and contested. The lifecycle therefore evolves through repeated interactions among technological capabilities, communicative responses, and social consequences, making generative AI's role inherently dynamic rather than fixed at any single stage.

Compared with a similar lifecycle review, Park and Nan (8), which primarily mapped the existing literature on generative AI and misinformation, the present framework makes several additional conceptual contributions. First, it not only organizes prior findings around four interrelated functions (i.e., production, detection, correction, and impact) but also specifies the mechanisms through which generative AI operates within each domain, including hallucination, amplification of falsehoods, adversarial vulnerability, deepfakes, fact-checking and pattern recognition, tailored correction, conversational rebuttal, and the persuasive affordances of fluency, narrative, and personalization. Second, the framework theorizes these domains as dynamically interconnected rather than merely co-occurring, emphasizing feedback pathways through which detection, correction, and downstream impacts can reshape subsequent misinformation production and response strategies. Finally, the present review adopts a health-specific lens that foregrounds mechanisms and consequences that may be especially salient in health communication, including risk perception, clinical and institutional trust, health behavior, epistemic uncertainty, health inequities, and system strain.

### Implications for health communication research and practice

The analysis presented here suggests that generative AI is a structural feature of the contemporary health information environment. As such, its implications extend beyond misinformation content to the theories, methods, and institutional practices that guide health communication.

First, generative AI challenges foundational assumptions about sources, credibility, and expertise. Much of health communication theory distinguishes between institutional, interpersonal, and media sources, often treating expertise and authority as socially anchored properties. AI systems complicate this framework by producing expert-styled communication without stable social identity, professional accountability, or institutional location. This shift calls for renewed theoretical work on *machine-mediated credibility*, epistemic authority, and how individuals assign trust when traditional source cues are absent or ambiguous (9, 41). Research must move beyond treating AI as a channel and instead conceptualize it as an actor-like component in persuasive processes.

Second, the features of AI-generated misinformation such as fluency, narrative coherence, and dynamic personalization require refinement of models of persuasion and misinformation processing. These systems operationalize message tailoring and narrative construction at a level of scale and responsiveness not previously available. This raises questions about how established constructs such as elaboration, perceived relevance, and causal reasoning operate when messages are continuously adapted within interaction rather than delivered as fixed texts (16, 62). Health communication research must therefore address persuasion in *interactive, generative environments*, where exposure is iterative and co-constructed rather than one-directional.

Third, the dual role of AI as both misinformation producer and mitigation tool calls for integrative research designs that examine production, detection, correction, and impact as interdependent processes. Traditional research often isolates misinformation exposure or correction effects in controlled settings. In AI-mediated environments, however, misinformation and correction can be generated by the same systems, and users may encounter both within a single conversational thread. This dynamic necessitates more ecological and systems-oriented approaches, including longitudinal designs, human-AI interaction studies, and analyses of how users navigate sequences of AI-generated claims and counterclaims.

Finally, the global and cross-cultural scalability of generative AI underscores the need for comparative and equity-oriented research. Because AI systems exhibit uneven performance across languages, cultural contexts, and levels of health literacy, their influence on misinformation may amplify existing disparities. Health communication research must therefore integrate perspectives from global health, digital inequality, and cultural communication to understand how AI-mediated information flows affect vulnerable populations differently.

For public health communication professionals, the findings suggest that AI-generated misinformation cannot be addressed solely through traditional content correction. Public health communication must adapt to an environment where misleading information can be produced at scale, personalized in real time, and presented with the stylistic features of expertise.

First, monitoring and response infrastructures will need to evolve. Surveillance of health misinformation must incorporate AI-generated content, including text, images, and synthetic audiovisual media. This requires technical capacity alongside communication expertise, as well as cross-platform and multilingual coordination.

Second, correction strategies should be designed with AI's affordances in mind. Generative systems can support scalable, dialogic correction that aligns with evidence-based principles—providing clear explanations, alternative causal accounts, and interactive engagement rather than simple myth-vs.-fact rebuttals (43, 64). However, such systems should operate under human oversight and institutional guidance to ensure accuracy, ethical alignment, and transparency.

Third, source transparency alone is unlikely to suffice. Simply labeling content as AI-generated does not reliably reduce perceived credibility or sharing intentions. Instead, practitioners may need to emphasize *process transparency*, clarifying evidence sources, uncertainty, and institutional backing, while strengthening public understanding of how generative AI systems work and where their limitations lie.

Finally, trust-building remains central. Because AI systems can displace trust from human institutions, public health organizations must reinforce their own visibility, accountability, and relational engagement with communities. AI tools may be useful for extending reach, but they should complement—not replace—trusted human communicators, healthcare providers, and community networks.

For care providers and clinicians, the framework highlights the need to anticipate misinformation as part of routine patient communication. Patients may increasingly arrive with questions, concerns, or treatment preferences shaped by AI-generated content that appears authoritative, personalized, and difficult to distinguish from professionally vetted information.

First, clinicians should be prepared to assess not only what patients believe, but also where that information came from and how it was generated. Asking patients about the sources they consulted, including conversational AI systems, can help identify misunderstandings, uncover the reasoning underlying their concerns, and create opportunities for corrective dialogue without dismissing the patient's information-seeking efforts.

Second, effective correction in clinical encounters should go beyond simply labeling information as false. Providers may need to explain why a claim is misleading, offer a clear evidence-based alternative, and relate that explanation to the patient's specific circumstances. Because generative AI can tailor misinformation to individual fears, values, and prior beliefs, corrective communication may likewise need to be responsive to those concerns while maintaining clinical accuracy and appropriate boundaries.

Third, clinicians should be cautious about relying on AI tools without verification, particularly when using them for patient education, visit preparation, or follow-up communication. Generative systems may be useful for translating, simplifying, or tailoring health information, but outputs should be reviewed against authoritative sources and used in ways that preserve professional accountability.

Finally, the rise of generative AI may increase the importance of clinicians as trusted interpreters of health information. Rather than competing with AI as an information source, care providers can help patients evaluate the credibility, relevance, and uncertainty of AI-generated claims. Incorporating brief discussions of AI-generated health information into shared decision-making may therefore become an increasingly important component of clinical communication and misinformation management.

### Limitations of the current evidence base

Although evidence on generative AI and health misinformation is expanding rapidly, the research landscape remains uneven. Much of the empirical work relies on laboratory studies, short-term experiments, or controlled prompt evaluations of model outputs. These approaches are essential for establishing baseline capabilities and risks, but they may not fully capture how AI-generated misinformation functions in naturalistic settings where exposure is repeated, multimodal, socially embedded, and shaped by platform dynamics. Moreover, models evolve quickly, meaning findings about specific systems may become outdated as architectures, safeguards, and usage patterns change.

There are also important gaps in population coverage. Much existing research focuses on English-language outputs and users in high-resource settings. Given documented disparities in model performance across languages and cultural contexts, the effects of AI-generated misinformation in low-resource settings remain insufficiently understood. Similarly, vulnerable populations, such as individuals with limited health literacy, constrained healthcare access, or high baseline mistrust of institutions, may experience AI-mediated information environments differently, yet are underrepresented in current studies.

Another key limitation of the current evidence base is that the findings are necessarily grounded in studies examining earlier generations of widely used generative AI systems, such as GPT-3.5/4 and early versions of comparable models. Given the rapid pace of technological development, model capabilities are evolving quickly, with newer iterations demonstrating improvements in accuracy, reasoning, and safety features. As a result, the evidence synthesized here may not fully capture the performance of the most recent systems. Accordingly, the conclusions of this review should be interpreted within the context of a dynamic and continuously changing technological landscape, where both the risks and potential benefits of generative AI for health misinformation are likely to shift over time.

A further limitation of the current evidence base is that most studies examine generative AI in non-clinical, public-facing contexts, rather than in formal clinical decision-making settings. Consequently, these findings may not directly generalize to healthcare environments, where technologies are interpreted alongside clinical expertise, patient interaction, and multiple sources of medical data. In practice, clinicians integrate diverse evidence to mitigate risks such as false positives and false negatives, rather than relying on a single tool. By contrast, the literature reviewed here primarily reflects AI use as an informal information source, often without professional oversight. This distinction highlights an important boundary of the current evidence and a key direction for future research.

Another caveat is that some of the evidence informing the integrative framework was drawn from studies examining other forms of misinformation, including false or misleading political claims, general conspiracy theories, and tourism misinformation. The extent to which findings from these domains generalize to health misinformation warrants further scrutiny. For example, findings from political misinformation may not transfer directly to health contexts because the mechanisms underlying message processing and behavioral response may differ across domains. Health misinformation often involves heightened perceptions of personal risk, greater reliance on professional or clinical authority, and potentially more consequential outcomes for individuals. These features may moderate the effects of generative AI-enabled mechanisms such as personalization, narrative fluency, source cues, or corrective messaging.

Finally, causal chains linking AI-generated misinformation to long-term health behaviors and outcomes are still emerging. While evidence supports effects on knowledge, risk perceptions, and intentions, fewer studies have traced downstream consequences for sustained behavior or population-level health indicators. These gaps underscore the need for longitudinal, field-based, and cross-cultural research that situates AI within real-world communication ecologies.

## Conclusion

Generative AI is reshaping the health misinformation environment in ways that extend well beyond the production of false or misleading content. By integrating evidence across production, detection, correction, and impact, this review shows that generative AI occupies multiple, sometimes contradictory roles across a health misinformation lifecycle that is recursive, adaptive, and continuously evolving. The same capabilities that enable misinformation to be generated at scale, rendered more persuasive, and tailored to individual users can also support its detection and correction. These dual functions create new opportunities for public health communication while introducing challenges related to accuracy, trust, equity, oversight, and accountability. Addressing AI-mediated health misinformation will therefore require more than isolated technical or communication interventions; it will require coordinated strategies that recognize the interdependence of technological systems, human judgment, institutional practices, and social consequences. As generative AI becomes increasingly embedded in everyday health information seeking, understanding and managing these interactions will be essential to preserving the integrity of health information and supporting informed health decision-making.

## Footnotes

^1^The 10 key studies were selected based on their exclusive focus on health misinformation, use of empirical data, and representativeness of the relevant lifecycle domain.
